# Two-dimensional, conductive niobium and molybdenum metal–organic frameworks†
†Electronic supplementary information (ESI) available: Additional experimental details, additional characterization data (powder X-ray diffraction, NMR spectroscopy, X-ray absorption spectroscopy, infrared spectroscopy, UV-vis-NIR spectroscopy, magnetic susceptibility, cyclic voltammetry, TEM), and supplementary discussion. See DOI: 10.1039/d0sc02515a


**DOI:** 10.1039/d0sc02515a

**Published:** 2020-06-02

**Authors:** Michael E. Ziebel, Justin C. Ondry, Jeffrey R. Long

**Affiliations:** a Department of Chemistry , University of California , Berkeley , CA 94720 , USA . Email: jrlong@berkeley.edu; b Materials Sciences Division , Lawrence Berkeley National Laboratory , Berkeley , CA 94720 , USA; c Department of Chemical Engineering , University of California , Berkeley , CA 94720 , USA

## Abstract

The incorporation of second-row transition metals into metal–organic frameworks could greatly improve the performance of these materials across a wide variety of applications due to the enhanced covalency, redox activity, and spin–orbit coupling of late-row metals relative to their first-row analogues. Thus far, however, the synthesis of such materials has been limited to a small number of metals and structural motifs. Here, we report the syntheses of the two-dimensional metal–organic framework materials (H_2_NMe_2_)_2_Nb_2_(Cl_2_dhbq)_3_ and Mo_2_(Cl_2_dhbq)_3_ (H_2_Cl_2_dhbq = 3,6-dichloro-2,5-dihydroxybenzoquinone), which feature mononuclear niobium or molybdenum metal nodes and are formed through reactions driven by metal-to-ligand electron transfer. Characterization of these materials *via* X-ray absorption spectroscopy suggests a local trigonal prismatic coordination geometry for both niobium and molybdenum, consistent with their increased covalency relative to related first-row transition metal compounds. A combination of vibrational spectroscopy, magnetic susceptibility, and electronic conductivity measurements reveal that these two frameworks possess distinct electronic structures. In particular, while the niobium compound displays evidence for redox-trapping and strong magnetic interactions, the molybdenum phase is valence-delocalized with evidence of large polaron formation. Weak interlayer interactions in the neutral molybdenum phase enable solvent-assisted exfoliation to yield few-layer hexagonal nanosheets. Together, these results represent the first syntheses of metal–organic frameworks containing mononuclear niobium and molybdenum nodes, establishing a route to frameworks incorporating a more diverse range of second- and third-row transition metals with increased covalency and the potential for improved charge transport and stronger magnetic coupling.

## Abstract 2

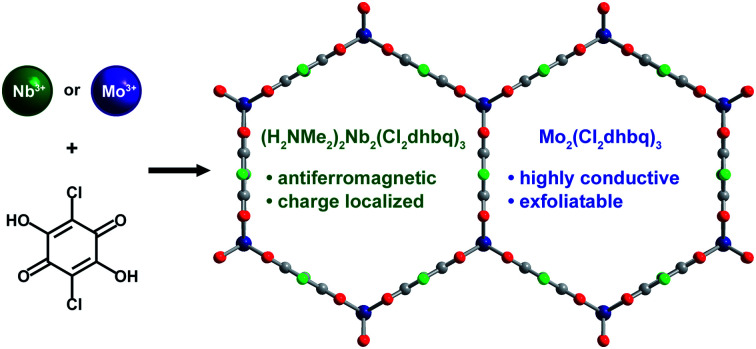
Incorporation of Nb and Mo into conductive metal–organic frameworks enables utilization of the enhanced covalency, redox activity, and spin–orbit coupling of late-row metals to improve the transport and magnetic properties of these materials.

## Introduction

Second- and third-row transition metals offer a diverse range of structural, electronic, and magnetic properties not accessible with first-row metals, including increased covalency in bonding, strong spin–orbit coupling, diverse coordination geometries, and access to a wide range of oxidation states.^1^ In solid-state materials, these properties can manifest as strong electronic correlations,^2^,^3^ large magnetic anisotropies,^4^,^5^ and enhanced electrochemical performance.^6^,^7^ Such properties inspire the development of metal–organic frameworks based on second- and third-row transition metals, but examples to date have been largely limited to frameworks generated from redox-inert d^0^ and d^10^ metals that engage in ionic bonding (Zr^4+^, Hf^4+^, and Cd^2+^),^8^–^10^ paddlewheel building units (*e.g.*, Mo_2_^4+^ and Ru_2_^4/5+^),^11^–^13^ and diamagnetic square-planar metals (*e.g.*, Pd^2+^ and Pt^2+^).^14^,^15^

Although the examples are limited, some second- and third-row transition metal coordination solids do exhibit phenomena consistent with the properties of second- and third-row metals. For example, one-dimensional chains of Pt_2_(dta)_4_I (dta^2–^ = dithioacetate) display metallic conductivity,^16^,^17^ while two- and three-dimensional frameworks based on Ru_2_-tcnq (tcnq = tetracyanoquinodimethane) linkages have been found to possess high magnetic ordering temperatures and large magnetic coercivities.^18^,^19^ However, frameworks incorporating square planar Pd^2+^, Pt^2+^, Ag^1+^, and Au^1+^ display lower conductivities than their Ni and Cu analogues, suggesting simple incorporation of second- and third-row metals does not guarantee enhanced performance.^14^,^15^,^20^ Expanding the synthetic scope of second- and third-row frameworks to new families of materials should help to identify design strategies that will utilize the unique electronic and magnetic properties of such heavy transition metals. In particular, targeting frameworks based on early second- and third-row metal ions (Nb, Mo, Ta, and W), which offer more diffuse orbitals and greater possible spin states than the late transition metals, is a promising strategy for the development of porous and guest-responsive magnets that exhibit both large magnetic anisotropy and magnetic polarization.

The scarcity of metal–organic frameworks based on these early second- and third-row transition metals can be attributed to a variety of synthetic challenges. First, strong coordination of ligands to second-row metals leads to slow kinetics of ligand exchange, which inhibits the reversible coordination necessary to form crystalline frameworks.^21^ Second, these metals tend to possess higher oxidation states, higher coordination numbers, and larger oxophilicities than their first-row counterparts, which can lead to formation of metal–oxo species under typical solvothermal synthesis conditions and complicate the synthesis of isostructural frameworks.^22^ Finally, common reagents used to prepare metal–organic frameworks featuring first-row transition metals may be inaccessible for the heavier transition metals, or exhibit entirely different structures with these metals. For example, the second-row metal acetates typically exist as dinuclear paddlewheels with strong metal–metal bonds,^23^ metal nitrates are only available with d^0^ and d^10^ ions, and metal–halide bonds are kinetically inert,^21^ complicating the mechanism of framework formation. Taken together, these challenges underscore the need for new synthetic strategies to develop metal–organic frameworks based on second- and third-row metal ions.

One potential route to the incorporation of second-row transition metals into framework materials is to use redox-active ligands and metal-to-ligand electron transfer to promote framework formation. For example, although the synthesis of vanadium(ii)-containing frameworks faces many of the same challenges as the formation of second-row transition metal frameworks,^24^ the reaction of VCl_2_ with chloranilic acid (H_2_Cl_2_dhbq) yields a crystalline honeycomb framework with an oxidized vanadium ion and a reduced ligand.^25^ Given that the reduction potentials of second-row metals are typically higher than those of the first-row metals,^26^ we rationalized that a similar approach could be used to access frameworks featuring second-row transition metals.

Herein, we report the synthesis and electronic properties of two-dimensional niobium– and molybdenum–chloranilate frameworks. While the two frameworks are isoelectronic and exhibit enhanced metal–ligand covalency relative to their first-row analogues, their electronic structures are vastly different, as determined through spectroscopic, magnetic, and electronic transport measurements. Specifically, the electronic structure of the Nb phase is highly localized, which results in a low electronic conductivity, while the Mo phase is highly conductive, likely due to the formation of large polaronic species. The neutral Mo phase is easily exfoliable in polar, organic solvents, enabling the isolation of few-layer nanosheets.

## Results and discussion

### Synthesis and structure

The majority of two-dimensional transition metal phases based on 2,5-dihydroxybenzoquinone and its functionalized derivatives are anionic frameworks that are synthesized using divalent metal precursors.^27^–^30^ With sufficiently reducing precursors featuring, for example, Ti^II^, V^II^, Cr^II^, or Fe^II^, metal-to-ligand electron transfer during synthesis can yield frameworks with tri- or tetravalent metal centers and partially or fully reduced ligands.^25^,^31^,^32^ Given the scarcity of mononuclear, divalent reagents available for the synthesis of analogous niobium and molybdenum frameworks, as well as the more reducing character of Nb^III^ and Mo^III^ relative to V^III^ and Cr^III^, we sought to synthesize materials of this type using the trivalent precursors NbCl_3_(DME) (DME = 1,2-dimethoxyethane) and [Mo(DMF)_6_](CF_3_SO_3_)_3_.^33^,^34^

**Fig. 1:**
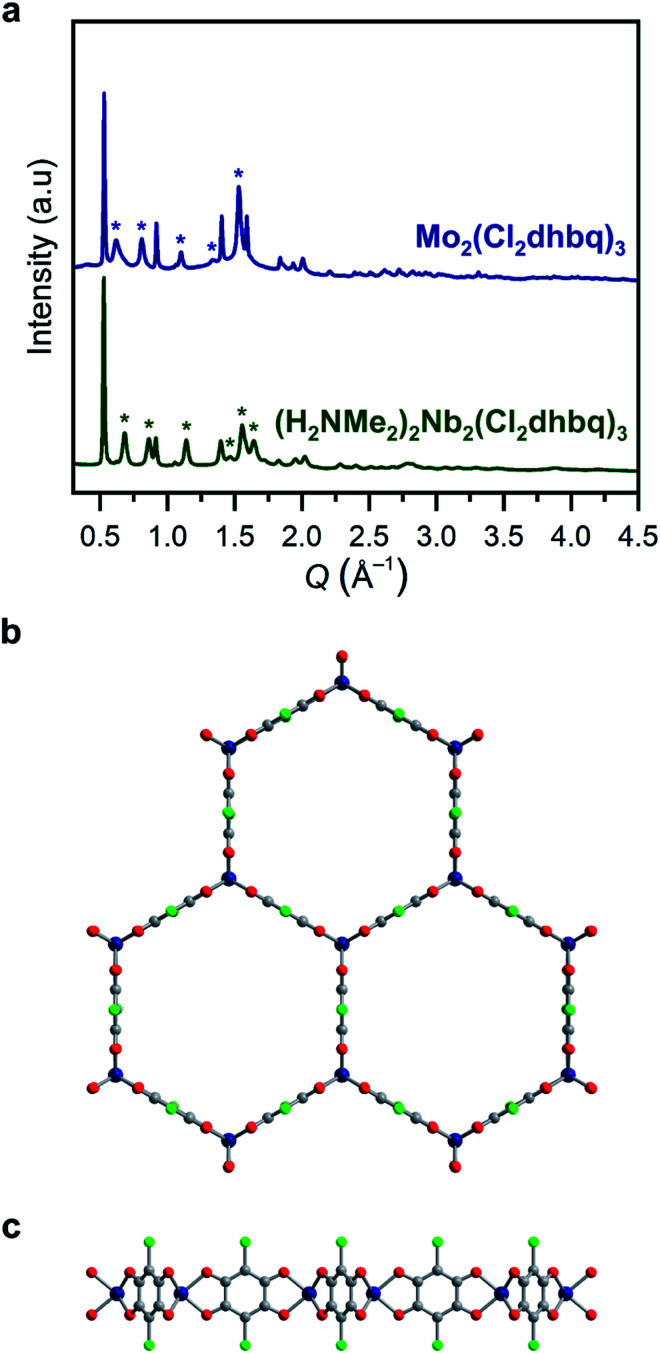
(a) Powder X-ray diffraction data for **1-Nb** and **2-Mo**. Unit cell indexing suggests honeycomb structures with the space group *P*3[combining macron]1*m*, similar to those reported for other metals.^25^,^31^ Le Bail refinement generates unit cell parameters of *a* = 13.743(6)/13.89(2) Å and *c* = 9.204(8)/10.38(4) Å for **1-Nb** and **2-Mo**, respectively. Stars correspond to prominent reflections with a *c* axis component. Model of the two-dimensional framework in **1-Nb** and **2-Mo** as viewed along (b) the *c* axis and (c) the *ab* plane. Dark blue, green, red, and gray spheres correspond to Nb/Mo, Cl, O, and C atoms, respectively. We note that this is a model structure generated by applying a trigonal prismatic geometry to previously reported structures, rather than a structure refined from powder X-ray diffraction. Some deviation from a perfect trigonal prismatic geometry may be present in the actual crystal structures.

The stoichiometric reaction of these reagents with H_2_Cl_2_dhbq in DMF yielded brick red and dark blue microcrystalline powders, respectively, with powder X-ray diffraction patterns consistent with the two-dimensional honeycomb phases [Nb_2_(Cl_2_dhbq)_3_]^*n*–^ (**1-Nb**) and [Mo_2_(Cl_2_dhbq)_3_]^*n*–^ (**2-Mo**) (Fig. 1 and S1†). Both phases show substantial peak broadening for reflections with a *c*-axis component, indicative of some degree of disorder in interlayer stacking or small domain sizes along the *c*-axis (Fig. S2 and S3†). Interestingly, crystalline **2-Mo** forms at room temperature, while a reaction temperature of 120 °C was necessary to form **1-Nb**, similar to conditions used for the titanium and vanadium frameworks. Indeed, the synthesis of frameworks containing second-row transition metals often require elevated temperatures to overcome the kinetic inertness of these metals.^11^,^12^ The formation of crystalline **2-Mo** at a much lower temperature supports the importance of the metal-to-ligand electron transfer in promoting framework formation. Notably, **1-Nb** is the first monometallic niobium metal–organic framework,^35^–^37^ and **2-Mo** is the first monometallic molybdenum framework that is not constructed from paddlewheel building units.^11^,^38^

If metal-to-ligand electron transfers occur exclusively through inner-sphere processes, the use of a trivalent precursor complex should yield charge-neutral frameworks of the general formula M_2_(Cl_2_dhbq)_3_, regardless of the final redox states of the metals and ligands. Charge-balancing cations in the pores of anionic chloranilate frameworks have been shown to promote ordered interlayer stacking over large length scales,^39^ and thus, the absence of charge-balancing cations in **1-Nb** and **2-Mo** could explain the broad peaks observed in their powder X-ray diffraction patterns. To test for the presence of alkylammonium cations, the frameworks were digested in D_2_O and the resulting solutions were analysed by ^1^H NMR spectroscopy (Fig. S4†). Only DMF was observed in the spectrum for digested **1-Mo**, confirming the formation of charge-neutral Mo_2_(Cl_2_dhbq)_3_·6DMF layers. However, peaks for both dimethylammonium cations and DMF were present in the spectrum of digested **1-Nb**, indicating that the parent framework carries an anionic charge. A combination of ^1^H NMR spectroscopy and elemental analysis data collected for the solvated and activated **1-Nb** materials indicate a formulation of (H_2_NMe_2_)_2_Nb_2_(Cl_2_dhbq)_3_·4.1DMF.

The anionic character of this material can likely be explained by the dinuclear, metal–metal bonded nature of the NbCl_3_(DME) precursor, which is more accurately represented as (DME)Cl_2_Nb(μ-Cl)_2_NbCl_2_(DME).^40^ If both Nb^III^ centers from each Nb_2_ unit were incorporated into the product, the resulting framework should be charge neutral; instead, we propose that, a one-electron inner-sphere, metal-to-ligand electron transfer occurs from each Nb^III^ center with concomitant cleavage of the Nb–Nb bond, such that only one Nb^III^ center in each dinuclear unit incorporates into the product, while the other acts purely as an electron donor. A complex mechanism of this type and the need to break metal–metal bonds may also explain the elevated synthesis temperature required for **1-Nb** relative to **2-Mo**. Interestingly, this extra electron transfer enabled by the metal–metal bond renders **1-Nb** isoelectronic to **2-Mo**, as well as to the previously reported vanadium congener. Here, we use the term isoelectronic to refer to the valence electron count of the framework, ignoring ligand redox activity. As discussed below, the actual distribution of electrons between metals and ligands, as well as the electron configuration at the metal centers, varies with metal identity among the three materials.

All structurally characterized transition metal–chloranilate frameworks possess a pseudo-octahedral coordination geometry around the metal center.^27^,^28^,^31^,^41^ While there are no structurally characterized, homoleptic niobium (tris)dioxolene compounds, all reported niobium tris(dithiolene) compounds possess a trigonal prismatic coordination geometry.^42^–^44^ Homoleptic molybdenum tris(dioxolene) complexes are reported with both octahedral and trigonal prismatic geometries,^45^,^46^ while all neutral molybdenum tris(dithiolenes) possess a trigonal prismatic geometry.^47^,^48^ These molecular analogues suggested the possibility of a trigonal prismatic coordination geometry in **1-Nb** and **2-Mo**. We note that this coordination geometry would not alter the symmetry or connectivity of the two-dimensional framework. However, the orientation of the chlorine substituents along the *c*-axis in this geometry may disrupt interlayer stacking, which could further explain the broad peaks in the diffraction patterns for these materials.

**Fig. 2:**
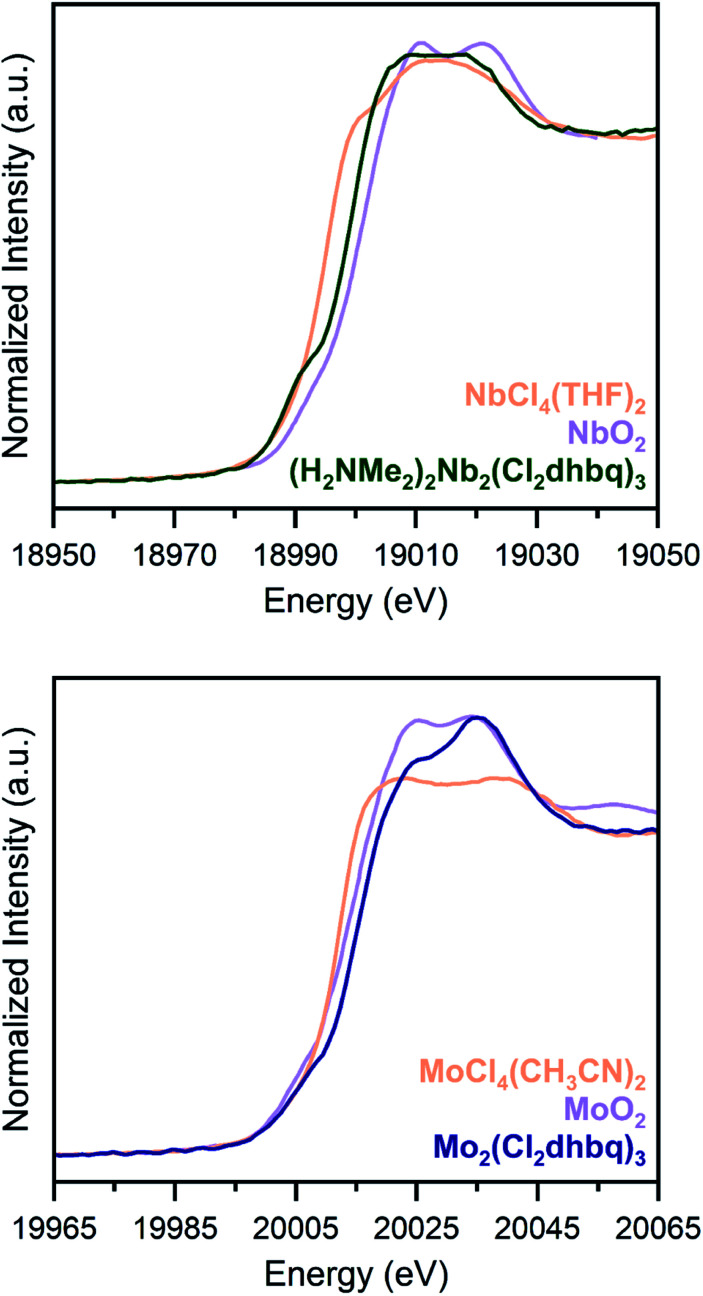
K-edge X-ray absorption spectra for **1-Nb** (top) and **2-Mo** (bottom), along with tetravalent metal oxides (orange) and molecular chloride complexes (purple), as reference compounds. Both frameworks display pre-edge features which are indicative of a trigonal prismatic geometry. Data for NbO_2_ and MoO_2_ are taken from ref. 34 and 53.

Due to substantial solvent disorder in the pores of **1-Nb** and **2-Mo** and reduction in framework crystallinity upon activation, structural refinement from powder X-ray diffraction data proved challenging. We therefore turned to Nb and Mo K-edge X-ray absorption spectroscopy (XAS) as a means of distinguishing between octahedral and trigonal prismatic coordination geometries in **1-Nb** and **2-Mo** (Fig. 2). The absence of inversion symmetry at the metal center in trigonal prismatic coordination enables 4d–5p mixing, which enhances the Laporte-forbidden 1s to 4d pre-edge transition.^49^ While the short core-hole lifetime in heavy elements leads to broadening of pre-edge features,^50^ the distinct pre-edge features observed for both **1-Nb** and **2-Mo** support a local trigonal prismatic geometry (Fig. S5†). While the slightly weaker pre-edge feature in **2-Mo** could potentially support a geometry intermediate between octahedral and trigonal prismatic, as has been observed in some anionic tris(dithiolene) complexes,^51^,^52^ we note that the pre-edge intensity for **2-Mo** is similar to that of trigonal prismatic 2H-MoS_2_.^53^

To assess if any of the early first-row transition metals possess a similar trigonal prismatic coordination geometry, we collected transition metal K-edge spectra for the Ti, V, and Cr congeners, (H_2_NMe_2_)_2_Ti_2_(Cl_2_dhbq)_3_, (H_2_NMe_2_)_2_V_2_(Cl_2_dhbq)_3_, and (H_2_NMe_2_)_1.5_Cr_2_(dhbq)_3_ (Fig. S6†).^25^ Among these three first-row metal materials, only the vanadium framework displays a high-intensity pre-edge feature suggestive of trigonal prismatic coordination. In neutral or monoanionic tris(dithiolene) complexes, this geometry is indeed favored due to the improved π-orbital overlap,^54^ and evidence of this coordination geometry for the vanadium framework is consistent with its highly covalent and delocalized electronic structure.^25^ As such, the trigonal prismatic coordination of **1-Nb** and **2-Mo** provides an indication of enhanced covalency for these materials relative to those incorporating most first-row metals.

### Electronic structure

Chloranilate frameworks synthesized with the early transition metals display a range of metal and ligand redox states. For example, (H_2_NMe_2_)_2_Ti_2_(Cl_2_dhbq)_3_ features Ti^IV^ centers with ligands in the trianionic and tetraanionic state, while (H_2_NMe_2_)_1.5_Cr_2_(dhbq)_3_ possesses Cr^III^ centers with dianionic and trianionic ligand states.^25^ Given the increased reducing power of second-row metals, we expected that a similar diversity of redox states could be accessible in **1-Nb** and **2-Mo**.

As an initial probe of the metal oxidation states in these materials, we analysed the rising edge portion of the K-edge X-ray absorption spectra (Fig. 2). The rising edge energy for **1-Nb** is 18999.6(2) eV, between the rising edge energies of NbCl_4_(THF)_2_ and NbO_2_, while the rising edge energy of **2-Mo** is 20015.3(2) eV, higher than the rising edge energies of both MoCl_4_(CH_3_CN)_2_ and MoO_2_.^36^,^55^ Because rising edge energies are highly dependent on the nature of the coordinated ligands, and because of the lack of molecular analogues for **1-Nb** and **2-Mo** with well-characterized oxidation states, absolute oxidation state assignment from XAS data alone is challenging. Metal–ligand covalency in these frameworks further complicates oxidation state assignment, as in the case of molecular dithiolene complexes.^56^ However, the rising edge energies of **1-Nb** and **2-Mo** are most consistent with Nb^IV^ and Mo^IV^, and these assignments are consistent with other spectroscopic and magnetic characterization of these frameworks, as discussed below. An extended discussion of oxidation state assignment from the X-ray absorption spectra can be found in the ESI. We note that, as in dithiolene complexes, metal–ligand covalency may limit the utility of formal oxidation state assignments beyond the purposes of electron counting.

The ligand redox states in **1-Nb** and **2-Mo** can be inferred from the metal oxidation states and the charge per formula unit in each framework. For **1-Nb**, an assignment of Nb^IV^ corresponds to ligand redox states of [NbIV2(Cl_2_dhbq^3–^)_2_(Cl_2_dhbq^4–^)]^2–^, which matches the charge distribution previously observed for the titanium analogue.^25^ For **2-Mo**, an assignment of Mo^IV^ would correspond to a ligand redox-state formulation of Mo^IV^(Cl_2_dhbq^2–^)(Cl_2_dhbq^3–^)_2_. However, a more rigorous analysis for both materials is possible using data from infrared spectroscopy and magnetic susceptibility measurements. For example, in metal–chloranilate frameworks, the vibrational frequency of the quinone stretching mode is diagnostic of the redox state of the ligand. The dianionic ligand typically appears between 1500 and 1530 cm^–1^, while the trianionic ligand typically appears between 1420 and 1450 cm^–1^.^57^–^59^ Mixed-valent ligands that are equivalent on the vibrational timescale appear as a single, broad peak outside of these ranges. For instance, the quinone frequency for the titanium phase appears at 1404 cm^–1^.^25^

**Fig. 3:**
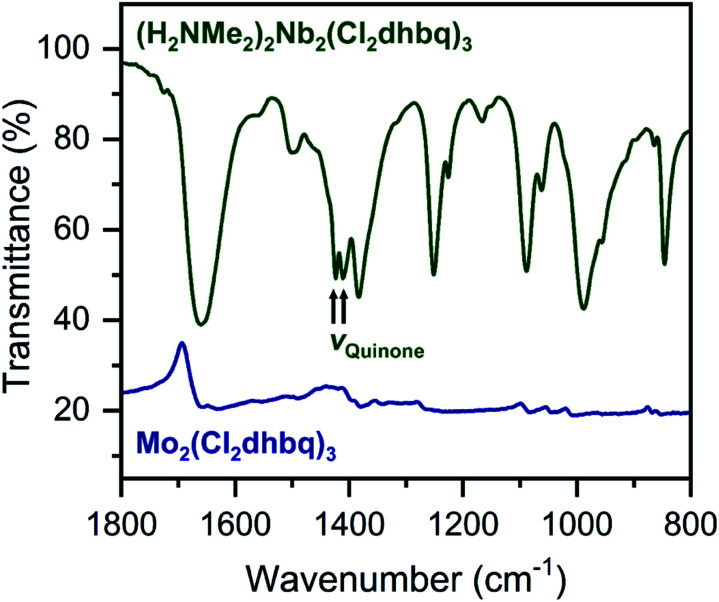
Portion of the infrared spectrum of **1-Nb** (dark green) and **2-Mo** (navy). We note that no offset is applied to the spectrum of **2-Mo**. Full spectra are shown in Fig. S7 and S9.†

Interestingly, in **1-Nb**, two strong features at 1424 and 1410 cm^–1^ are present in the frequency range expected for the quinone stretching mode (Fig. 3). We note that both features are present following removal of solvent from the framework pores, confirming that both vibrational modes are intrinsic to the framework (Fig. S7†). The vibrational mode at 1424 cm^–1^ is consistent with a localized Cl_2_dhbq^3–^ ligand, while the vibrational mode at 1410 cm^–1^ is more consistent with a valence-delocalized mixture of Cl_2_dhbq^3–^ and Cl_2_dhbq^4–^ ligands, as was observed for the titanium phase.^25^ The presence of two distinct vibrational modes has also been observed in the chromium and aluminum phases,^25^,^60^ where it was taken as an indication of redox-trapped dianionic and trianionic ligands. However, the presence of redox trapping in **1-Nb** is seemingly at odds with the observation of a vibrational mode corresponding to valence-delocalized redox states. This result may suggest a more complex electronic structure in which a portion of the ligands are redox-trapped, while others undergo rapid electron transfer on the vibrational timescale. The presence of redox-trapped ligands is further supported by the absence of a low-energy intervalence charge transfer feature in the near-infrared region (Fig. S8†). Higher energy transitions at 13600, 19700, and 30000 cm^–1^ can be assigned as charge transfer and ligand-centered excitations.

This unusual electronic structure may also explain the magnetic properties of **1-Nb**. Despite the expected presence of *S* = 1/2 Nb^IV^ ions and redox-trapped Cl_2_dhbq^3–^ ligands, **1-Nb** was found to be diamagnetic up to 300 K, except for a small Curie tail below 5 K (Fig. S10†). While this result could potentially support an oxidation assignment of [NbV2(Cl_2_dhbq^4–^)_3_]^2–^, such an electronic structure would be inconsistent with the XAS and vibrational spectroscopy data discussed above. Diamagnetism in **1-Nb** could also be consistent with band-like character, as is observed in NbS_2_,^61^ but such character would be inconsistent with the presence of multiple quinone stretching modes and the low electronic conductivity, as will be discussed further below. Instead, strong, local antiferromagnetic interactions between paramagnetic Nb^IV^ centers and Cl_2_dhbq^3–^ ligands could result in the observed diamagnetism in **1-Nb**, akin to the strong antiferromagnetic coupling observed in d^1^ tris(dithiolene) complexes with open-shell singlet ground states.^49^,^52^ Indeed, these interactions could potentially result in partial redox-trapping of the trianionic ligands. Given the apparent complexity of the electronic structure of **1-Nb**, charge ordering could give rise to more intricate magnetic structures (for example, strongly coupled [Nb_6_(Cl_2_dhbq^3–^)_6_]^6+^ hexagons), but powder X-ray diffraction data provide no evidence of a reduction in symmetry or of the presence of a supercell. We note that other coordination solids containing radical ligands have been shown to be diamagnetic, though this is most commonly attributed to band-like character in highly conductive materials, rather than through the antiferromagnetic interactions that we propose to be operative in **1-Nb**.^62^

The infrared spectrum of **2-Mo** features a broad absorbance extending across the mid-infrared region, which masks the expected vibrational modes for the ligand and prevents direct assignment of redox states (Fig. 2 and S9†). A similar feature was observed in the vanadium congener and was ascribed to large polaron formation.^25^,^63^ Here, we use the term large polaron to refer to an electron–phonon-coupled mid-gap state extending over multiple ligands. To probe for the presence of large polarons in **2-Mo**, we measured its dc magnetic susceptibility under a field of 0.1 T (Fig. S11 and S12†). A Curie–Weiss fit to the data from 25 to 250 K yielded values of *C* = 0.43 emu mol^–1^ K^–1^ and *θ* = –39 K (Fig. S11†). This value of *C* is much lower than the value of 0.75 emu mol^–1^ K^–1^ expected for two *S* = 1/2 Cl_2_dhbq^3–^ radicals and diamagnetic Mo^IV^ (given the expected low-spin electronic configuration of 
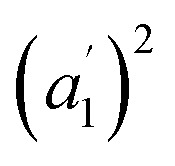
 for a trigonal prismatic coordination geometry). The low value of *C* and the relatively weak magnetic interactions suggested by the magnitude of *θ* support some degree of spin-quenching through large polaron formation. Additionally, the presence of an asymmetric, broad absorption feature extending across the near-infrared region (*ν*_max_ = 8760 cm^–1^) is consistent with the presence of strongly delocalized mixed-valent ligands (Fig. S8†). Finally, the higher energy of this transition relative to the Fe and Ti congeners is consistent with increased metal–ligand covalency, as discussed in a previously reported orbital model.^25^

### Transport properties

The vastly different electronic structures of **1-Nb** and **2-Mo** suggested that the two materials would display markedly different transport properties, despite having isoelectronic frameworks. Indeed, the electronic conductivity of **1-Nb** at 298 K is only 1.7(4) × 10^–6^ S cm^–1^, the lowest reported value for any anilate-based framework exhibiting metal-to-ligand electron transfer (Fig. S13†). Such a low conductivity is consistent with the absence of an absorbance feature at low energies. Interestingly, the conductivity of **1-Nb** is nearly two orders of magnitude lower than that of the Cr congener, in which all ligands are redox trapped on the vibrational timescale.^25^ This is surprising, as the presence of a valence-averaged quinone stretching mode suggests some degree of electron delocalization. Consequently, the very low conductivity of **1-Nb** may provide further support for local charge ordering that inhibits long-range charge transport. In contrast, **2-Mo** displays a much higher conductivity of 1.8(3) × 10^–2^ S cm^–1^ at 298 K, which is consistent with the proposed large polaronic character (Fig. S13†). This value is among the highest for any two-dimensional transition metal–anilate frameworks, with the current record of 0.45 S cm^–1^ being held by (H_2_NMe_2_)_2_V_2_(Cl_2_dhbq)_3_.^25^,^64^

The isoelectronic nature of **1-Nb**, **2-Mo**, and the previously reported vanadium phase enables an assessment of the importance of metal oxidation state, metal electron count, and ligand redox state on the transport properties of these materials. While the frameworks in all three materials have identical valence electron counts, their electronic structures are distinct. For example, **1-Nb** and **2-Mo** both contain tetravalent metal centers, but Nb^IV^ is paramagnetic while Mo^IV^ is diamagnetic, which may be important in explaining the electron localization in **1-Nb**. The redox states of the ligands are also different in the two phases, although from the present data, it is not clear what effect this difference may have on the distinct electronic properties of the materials. The electronic structure of **2-Mo** is more similar to that of the vanadium phase, and the electronic delocalization observed in these phases is also comparable. While the metal oxidation states and framework charges differ between the two materials, both possess d^2^ metal centers with a mix of dianionic and trianionic ligands. Based on magnetic data, vanadium likely possesses an *S* = 1 3d^2^ configuration,^25^ rather than the *S* = 0 4d^2^ configuration of **2-Mo**. From the above analysis, no apparent trends emerge that would uniformly explain the different transport properties of these materials. DFT calculations of the electronic structures of these three phases may provide more insights into the origins of charge localization in **1-Nb** and charge delocalization in **2-Mo**.

### Redox chemistry

One particularly interesting aspect of quinoid-based materials is the opportunity to alter their electronic structure and transport properties through post-synthetic reduction or oxidation.^32^,^64^ We expected that the increased covalency provided by second-row transition metal ions and the diverse range of accessible oxidation states available for these metals could lead to a large number of accessible redox states in **1-Nb** and **2-Mo**.^65^ Slow-scan cyclic voltammetry was used as an initial probe of the accessible reductions and oxidations in these frameworks. Surprisingly, **1-Nb** displays no fully reversible redox processes (Fig. S14†). Specifically, no reductive processes are observed down to 2.5 V *vs.* Li^0/+^, and poorly reversible oxidative processes are observed up to 3.55 V *vs.* Li^0/+^. Subsequent scans to lower and higher potentials revealed what may be quasi-reversible processes, although it is possible that these features correspond instead to decomposition products. In contrast, **2-Mo** displays a quasi-reversible reduction with *E*_1/2_ = 3.17 V *vs.* Li^0/+^, in addition to partially reversible oxidative features above 3.4 V *vs.* Li^0/+^ (Fig. S15†). These oxidative features are particularly interesting, as they represent anion insertion processes to generate a cationic framework. This cationic character may limit the stability of the framework to oxidation, which could explain the poor reversibility of these processes.

The limited stability of **1-Nb** and **2-Mo** to post-synthetic redox chemistry is surprising, given that the first-row transition metal analogues all show reversible electrochemical processes.^65^,^66^ The second-row analogues may be more susceptible to changes in coordination environment upon framework reduction or oxidation, which could limit the reversibility of these processes. As an initial assessment of the structural stability of **2-Mo** to oxidation, we carried out chemical oxidation of the framework with ferrocenium hexafluorophosphate. While full electronic characterization of the oxidized material lies outside the scope of this report, powder X-ray diffraction data suggests retention of the two-dimensional honeycomb structure (Fig. S16†). The narrower peak width of the *c*-axis reflections relative to the neutral framework is consistent with reduced stacking disorder upon anion insertion. Further studies of the structural and electronic effects of reduction and oxidation in **2-Mo**, and the origin of poor electrochemical reversibility in **1-Nb** and **2-Mo**, are underway.

### Exfoliation of **2-Mo**

**Fig. 4:**
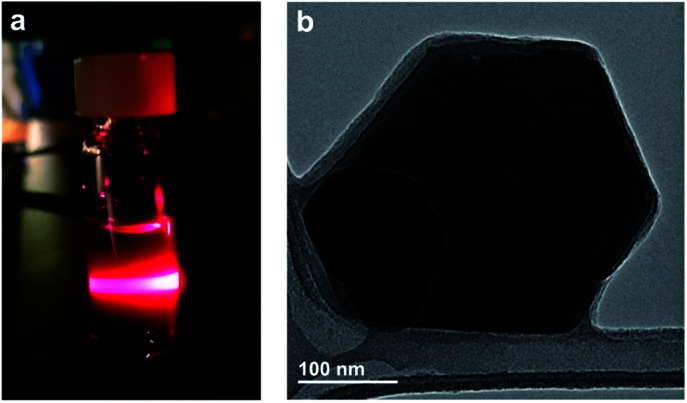
(a) Photograph of scattered 635 nm light from a suspension of **2-Mo** in DMF demonstrating the Tyndall effect. (b) TEM image of a few-layer nanosheet of **2-Mo**. Additional images are shown in Fig. S18.†

As discussed above, most two-dimensional frameworks based on 2,5-dihydroxybenzoquinone and its derivatives are anionic with charge-balancing cations situated within the pores or between two-dimensional layers. Despite the presence of these cations, nanosheets of iron-based materials have been reported with thicknesses down to seven monolayers.^67^ The charge neutrality of **2-Mo**, however, suggests exfoliation of this material may be possible, potentially down to the monolayer limit. Indeed, simply soaking powders of **2-Mo** in DMF at room temperature yielded blue colloidal suspensions that display the Tyndall effect, suggesting a particle size in the range of 50–1000 nm. Similar colloidal suspensions could also be generated in acetonitrile, although this required agitation of the powders *via* sonication (Fig. 4 and S17†).

Transmission electron microscopy (TEM) images of diluted, dropcast DMF suspensions revealed nanosheets with well-defined edges, hexagonal shapes, and edge lengths of 100 to 250 nm (Fig. 4b), whereas images of dropcast acetonitrile suspensions revealed substantial particle aggregation (Fig. S18†). Attempts to characterize these nanosheets using electron diffraction were unsuccessful, likely due to a combination of air exposure, electron beam damage, and reduced crystallinity from solvent loss under high vacuum. The TEM images obtained here suggest all nanosheets contain multiple framework monolayers; however, the ease of solvent-assisted exfoliation observed here implies that further optimization of the wet exfoliation procedure, or combining wet exfoliation with mechanical exfoliation, may generate monolayers of **2-Mo**. Currently, the small size of these nanosheets makes further characterization of the exfoliated materials challenging; attempts to improve the crystallite size of **2-Mo** through higher temperature and layered-solution syntheses are now underway. Finally, these results suggest that oxidation of anionic chloranilate frameworks to afford neutral frameworks could represent an improved approach for realizing monolayers of such materials.

## Conclusions

The foregoing results demonstrate that the use of metal-to-ligand electron transfer can facilitate the synthesis of new, redox-active metal–organic frameworks containing early second-row transition metals. Characterization of (H_2_NMe_2_)_2_Nb_2_(Cl_2_dhbq)_3_ and Mo_2_(Cl_2_dhbq)_3_*via* X-ray absorption spectroscopy suggested a local trigonal prismatic metal coordination, consistent with the increased covalency of second-row transition metals relative to their first-row analogues. Despite being isoelectronic, these materials possess distinct electronic structures, as demonstrated through spectroscopic and magnetic characterization. The niobium phase is diamagnetic and poorly electronically conductive, likely due to strong metal–ligand coupling and partially redox-trapped ligands, respectively, while the molybdenum phase was found to display substantial valence delocalization and high electronic conductivity. Critically, these results highlight that increased covalency arising from the use of second-row transition metals can result in strong magnetic interactions or improved charge transport in metal–organic materials, although these properties may be in competition with one another. The synthesis of the tantalum and tungsten analogues of these frameworks could provide further insights into the effects of enhanced metal–ligand covalency on the magnetic and charge transport properties of coordination solids. Further efforts will focus on the synthesis of new frameworks containing second-row metals with larger spins, in which strong magnetic coupling, such as that hypothesized to occur in **1-Nb**, could be used to achieve high magnetic ordering temperatures.

## Experimental methods

### Synthesis of materials

Unless otherwise specified, all syntheses and subsequent manipulations of purified materials were carried out under rigorous air- and water-free conditions, using Schlenk techniques or an Ar-atmosphere MBraun glovebox. The solvents *N*,*N*-dimethylformamide, acetonitrile, tetrahydrofuran, diethyl ether, and dichloromethane were dried using a commercial solvent purification system designed by JC Meyer Solvent Systems and were stored over 4 Å (DMF, CH_3_CN, THF, Et_2_O) or 3 Å (CH_2_Cl_2_) molecular sieves prior to use. Propylene carbonate was dried over melted sodium metal and distilled prior to use. The compound Mo(CO)_6_ (98%) was purchased from Sigma Aldrich and sublimed prior to use. The compounds NbCl_3_(DME),^33^ MoCl_4_(CH_3_CN)_2_,^68^ Ti(acac)_3_,^69^ (H_2_NMe_2_)_2_Ti_2_(Cl_2_dhbq)_3_, (H_2_NMe_2_)_2_V_2_(Cl_2_dhbq)_3_, and (H_2_NMe_2_)_1.5_Cr_2_(dhbq)_3_,^25^ were synthesized according to previously reported procedures. All other reagents were purchased and used as received: triflic acid (99.5%, Oakwood Chemical), triflic anhydride (99.5%, Oakwood Chemical), chloranilic acid (98%, Alfa Aesar), and Cr(acac)_3_ (97%, Sigma Aldrich). Compositional C, H, N, and S analyses were obtained from the Microanalytical Laboratory at the University of California, Berkeley.

#### Synthesis of Mo(DMF)_6_(CF_3_SO_3_)_3_

This compound was synthesized through a modified procedure for the synthesis of Mo(CF_3_SO_3_)_3_.^34^ Triflic acid (40 mL) and triflic anhydride (15 mL) were added to an oven-dried Schlenk flask and heated to 100 °C under an argon atmosphere to remove residual water. The solution was then degassed with three freeze–pump–thaw cycles, and freshly sublimed Mo(CO)_6_ (3.00 g, 11.4 mmol) was added under a positive pressure of argon. The reaction was stirred and heated at reflux for 18 h, and the resulting solid was allowed to settle to the bottom of the flask. The solvent was decanted, and dry, degassed diethyl ether (30 mL) was added to the flask. **Caution**: *The addition of diethyl ether to residual triflic acid is highly exothermic, and rapid addition can cause the solvent to boil*. This process was repeated three more times to remove residual triflic acid. The remaining solid was dried under reduced pressure at 100 °C for 3 h, after which it was dissolved in the minimal amount of DMF. Addition of diethyl ether to this solution precipitated the product as a bright yellow powder, which was collected by filtration and dried under reduced pressure. Yield: 5.1 g (45%). Analysis calcd. For C_21_H_42_F_9_MoN_6_O_15_S_3_: C, 25.7; H, 4.3; N, 8.6; S, 9.8. Found: C, 26.3; H, 4.4; N, 8.8; S, 9.8.

#### Synthesis of (H_2_NMe_2_)_2_Nb_2_(Cl_2_dhbq)_3_·4.1DMF

In an argon glovebox, NbCl_3_(DME) (15 mg, 0.052 mmol), chloranilic acid (16 mg, 0.076 mmol), and DMF (5 mL) were added to a 20 mL scintillation vial. The resulting blue solution was heated at 120 °C for 18 h. The reaction was allowed to cool, and the resulting red solid was collected by filtration. The crude product was soaked in 5 mL of DMF at room temperature for 24 h, and was then recollected by filtration. This was repeated two more times, after which the solid was dried at room temperature under reduced pressure for 2 h. For characterization, several independent batches were combined and thoroughly mixed. Yield: 16 mg (32%). Analysis calcd. For C_34.3_H_44.7_Cl_6_N_6.1_Nb_2_O_16.1_: C, 34.4; H, 3.8; N, 7.1. Found: C, 34.7; H, 3.7; N, 6.7. IR (solid-ATR): 3340 (w), 3167 (w), 3084 (w), 2931 (m), 2858 (m), 2805 (w), 2773 (w), 1660 (*vs.*), 1500 (m), 1424 (s), 1410 (s), 1384 (*vs.*), 1252 (s), 1226 (m), 1167 (w), 1088 (s), 1062 (m), 989 (*vs.*), 956 (w), 864 (w), 846 (s), 658 (s), 625 (m), 584 (m), 543 (*vs.*) cm^–1^.

To confirm the assignment of two dimethylammonium cations per formula unit, elemental analysis was collected on an activated framework. The DMF-solvated framework was soaked in successive aliquots of acetonitrile (2 × 10 mL), THF (2 × 10 mL), and CH_2_Cl_2_ (3 × 10 mL) for 12 h each wash, and with a filtration between each solvent exchange. The CH_2_Cl_2_-solvated framework was then activated at 120 °C under reduced pressure, transferred to a borosilicate tube capped with a Micromeritics TranSeal, and further activated for 24 h at 120 °C on a Micromeritics ASAP 2420 system to yield a black solid. We note that the color change observed upon activation is fully reversible, and soaking activated **1-Nb** in DMF restores the original red color. Analysis calcd. For C_22_H_16_Cl_6_N_2_Nb_2_O_12_: C, 29.4; H, 1.8; N, 3.1. Found: C, 29.6; H, 1.9; N, 3.3. IR (solid-ATR): 3079 (br), 2782 (w), 1699 (w), 1650 (w), 1557 (w), 1423 (s), 1409 (s), 1252 (s), 1164 (w), 988 (s), 959 (sh), 848 (s), 622 (w), 519 (*vs.*) cm^–1^.

#### Synthesis of Mo_2_(Cl_2_dhbq)_3_·6DMF

Solid Mo(DMF)_6_(CF_3_SO_3_)_3_ (225 mg, 0.229 mmol), chloranilic acid (75 mg, 0.36 mmol), and DMF (10 mL) were added to a 20 mL scintillation vial. The solution was left at room temperature for 24 h, and the resulting blue-black precipitate was collected by filtration. The crude product was soaked in 5 mL of DMF at room temperature for 24 h, and was then recollected by filtration. This was repeated two more times, after which the solid was dried at room temperature under reduced pressure for 2 h until it became a free flowing powder. We note that due to the rapid exfoliation of **2-Mo** in DMF, supernatant solutions remain colored even after repeated DMF washes. Yield: 166 mg (29%). Analysis calcd. For C_36_H_42_Cl_6_Mo_2_N_6_O_18_: C, 34.5; H, 3.4; N, 6.7. Found: C, 34.8; H, 3.6; N, 6.7.

#### Chemical oxidation of **2-Mo**

Solid **2-Mo** (15 mg, 0.012 mmol), Fe(Cp)_2_(PF_6_) (4.3 mg, 0.013 mmol), and DMF (2 mL) were added to a 4 mL scintillation vial, and the mixture was stirred for 24 h. The resulting black solid was collected by filtration and washed with successive aliquots of DMF (2 × 3 mL). The light yellow-green color of the filtrate suggested reduction of Fe(Cp)_2_^+^ to Fe(Cp)_2_, indicating at least partial oxidation of **2-Mo**.

#### Preparation of colloidal suspensions of **2-Mo**

DMF suspensions of **2-Mo** were prepared by soaking **2-Mo** (10 mg) in DMF (4 mL) at room temperature for 72 h. Acetonitrile suspensions were prepared by soaking **2-Mo** in CH_3_CN (4 mL), then agitating the suspension with a horn sonicator for 15 min. For both suspensions, residual solids were then removed *via* centrifugation, and the colloidal suspension was decanted from the solid. These suspensions were then further diluted with 10 mL of DMF or CH_3_CN.

### Powder X-ray diffraction

Microcrystalline powder samples of **1-Nb**, **2-Mo**, or oxidized **2-Mo** (1–2 mg) were loaded into 1.0 mm boron-rich glass capillaries inside an Ar-atmosphere glovebox, and the capillaries were flame sealed. High-resolution powder X-ray diffraction data were collected at beamline 17-BM at the Advanced Photon Source (APS) at Argonne National Laboratory. Diffraction patterns were collected at room temperature with a wavelength of 0.45118 (**1-Nb**) or 0.45236 (**2-Mo** and oxidized **2-Mo**) Å.

### ^1^H NMR spectroscopy

Microcrystalline samples of **1-Nb** or **2-Mo** (5 mg) were dissolved in D_2_O and sonicated briefly to ensure complete dissolution. Acid and heat were avoided during the digestion procedure to limit the decomposition of DMF to dimethylamine. ^1^H NMR spectra were recorded at room temperature on a Bruker AV-300 spectrometer and referenced to residual H_2_O (*δ* = 4.79 ppm).

### X-ray absorption spectroscopy

Powders of **1-Nb**, **2-Mo**, NbCl_4_(THF)_2_, and MoCl_4_(CH_3_CN)_2_ (5 mg) were spread over Kapton tape and sealed between two layers of polypropylene tape with the edges coated in Torr Seal® low vapor pressure epoxy. The Nb and Mo K-edge spectra were collected at beamline 10-BM of the Materials Research Collaborative Access Team (MRCAT) at the Advanced Photon Source at Argonne National Laboratory. Data were collected at room temperature in fluorescence mode. Monochromator energies were aligned by collecting K-edge spectra of a Nb or Mo reference foil.

Samples of (H_2_NMe_2_)_2_Ti_2_(Cl_2_dhbq)_3_, Ti(acac)_3_, (H_2_NMe_2_)_2_V_2_(Cl_2_dhbq)_3_, (H_2_NMe_2_)_1.5_Cr_2_(dhbq)_3_, and Cr(acac)_3_ were loaded onto Kapton tape on an aluminium sample holder, and sealed within a Mylar bag to prevent air exposure over the course of data collection. The Ti, V, and Cr K-edge X-ray absorption spectra were collected at beamline 10.3.2 of the Advanced Light Source at Lawrence Berkeley National Laboratory with the storage ring operating at 500 mA and 1.9 GeV. Data were collected at room temperature in fluorescence mode using a seven-element Ge solid-state detector and XIA electronics. Data collection was performed with the beam defocused by 10 mm to reduce beam damage during data collection. Monochromator energies were calibrated using a Ti, V, or Cr foil, and an internal *I*_0_ glitch present in all spectra was used to calibrate individual spectra. Data were processed using custom LabView Software available at the beamline.

Data reduction, baseline correction, and normalization for all spectra were performed using Athena, a part of the Demeter software package.^70^

### Infrared spectroscopy

Infrared spectra were collected on a PerkinElmer Avatar Spectrum 400 FTIR spectrometer equipped with a Pike attenuated total reflectance accessory. A custom glovebag attachment was used to perform measurements on air-sensitive samples under a dinitrogen atmosphere.

### UV-vis-NIR spectroscopy

UV-vis-NIR diffuse reflectance spectra were collected with a CARY 5000 spectrophotometer interfaced with Varian WinUV software. Spectra were collected in a Praying Mantis air-free diffuse reflectance cell using BaSO_4_ for the background measurement and as the non-absorbing matrix. The Kubelka–Munk conversion (*F*(*R*) *vs.* wavenumber) of the raw diffuse reflectance spectrum (*R vs.* wavenumber) was obtained by applying the formula *F*(*R*) = (1 – *R*)^2^/2*R*.

### Magnetic measurements

Samples were prepared by adding crystalline powders of **1-Nb** or **2-Mo** to a 5 mm inner diameter quartz tube containing a raised quartz platform. Solid eicosane was added to cover the sample to prevent crystalline torqueing and improve thermal contact between the sample and the cryostat. The tubes were fitted with sealable Teflon adapters, evacuated, and flame-sealed under static vacuum. Following flame sealing, the solid eicosane was melted in a water bath held at 40 °C. Magnetic susceptibility measurements were performed using a Quantum Design MPMS2 SQUID magnetometer. For **1-Nb** and **2-Mo**, dc magnetic susceptibility measurements were collected in the temperature range 2–300 K under applied magnetic fields of 0.1 and 1 T. Above 250 K, the very low moment of **2-Mo** resulted in substantial noise in the data. Contributions from the sample holder were background subtracted. Diamagnetic corrections were applied to the data using Pascal's constants to give *Χ*_D_ = –0.00055248 emu mol^–1^ (**1-Nb**), *Χ*_D_ = –0.00056848 emu mol^–1^ (**2-Mo**) and *Χ*_D_ = –0.00024306 emu mol^–1^ (eicosane). For **1-Nb**, two independently synthesized samples confirmed the diamagnetism of the sample. Minimal temperature independent paramagnetism was observed (*Χ*_TIP_ < 10^–4^ emu mol^–1^).

### Conductivity measurements

Two-point pressed-pellet conductivity measurements were conducted in a home-built, two-electrode screw cell described previously.^32^ Pellets of **1-Nb** or **2-Mo** were pressed between two copper rods with diameters of roughly 0.05 cm^2^. Sample thicknesses were measured with a caliper and were within the range 200 to 500 μm. *I*–*V* profiles were collected at room temperature over a voltage window of ±1 V using a Bio-Logic VMP-3 multipotentiostat. All measured samples displayed ohmic behavior in this window. Conductivity was calculated according to Ohm's Law. For both materials, reported conductivities represent average conductivity values for three independent pellets.

### Solid-state electrochemical characterization

Electrochemical data were collected in a three-electrode cell constructed using a 1/2′′ Swagelok PFA union tee and Ti current collectors. Counter and reference electrodes were smeared with Li metal and polished to a mirror finish. Suspensions of **1-Nb** (8 mg) or **2-Mo** (10 mg), Super P conductive carbon (2 mg), and polyvinylidene fluoride (2 mg) in 1 mL THF were sonicated for 15 min and dropcast onto carbon cloth electrodes. Quartz fibre soaked in a 0.1 M LiBF_4_/propylene carbonate solution was used as the separator. Cyclic voltammograms were collected at 50 μV s^–1^ using a Bio-Logic VMP-3 multipotentiostat.

### Transmission electron microscopy

A single drop from the DMF or CH_3_CN colloidal suspension of **2-Mo** was cast on a lacey-carbon-coated Au TEM grid (Ted Pella, Inc.) and allowed to evaporate at room temperature inside an Ar atmosphere glovebox. The samples were transferred from the glovebox in a sealed vial, quickly loaded into an FEI single tilt sample holder, and inserted into the microscope antechamber with less than 1 min of total air exposure. TEM images were obtained with a FEI Tecnai T20 S-TWIN TEM operating at 200 kV with a LaB_6_ filament (column pressure 8.8 × 10^–8^ torr). Images were collected using a Gatan Orius SC200 camera and analysed using ImageJ.

## Conflicts of interest

There are no conflicts to report.

## Supplementary Material

Supplementary information
