# Supplementary material for: Two-dimensional, conductive niobium and molybdenum metal–organic frameworks
Source: Chem Sci. 2020 Jun 2;11(26):6690–700. doi: 10.1039/d0sc02515a (PMC7481840; doi:10.1039/d0sc02515a)
Supplement: Supplementary file 1 [file SC-011-D0SC02515A-s001.pdf]

Electronic Supplementary Material (ESI) for Chemical Science.
This journal is © The Royal Society of Chemistry 2020

Supplementary Information For:

Two-Dimensional, Conductive Niobium and Molybdenum Metal–Organic Frameworks

Michael E. Ziebel,[ab] Justin C. Ondry,[a] and Jeffrey R. Long*[abc]

[a]Department of Chemistry, University of California, Berkeley, California 94720, USA

[b]Materials Sciences Division, Lawrence Berkeley National Laboratory, Berkeley, California 94720 USA

[c]Department of Chemical Engineering, University of California, Berkeley, California, 94720, USA

Table of Contents

Powder X-ray Diffraction S-2

Spectroscopic Characterization S-5

Magnetic Characterization S-9

Electronic Conductivity S-10

Redox Activity of 1-Nb and 2-Mo S-11

Exfoliation of 2-Mo S-13

References S-14

Powder X-ray Diffraction

For all samples, a standard peak search, followed by indexing via single-value decomposition, as implemented in TOPAS-Academic v4.1,[1,2] enabled space group assignment and determination of approximate unit cell parameters. Precise unit-cell dimensions were obtained through a structureless Le Bail refinement using the free software FOX.[3,4]

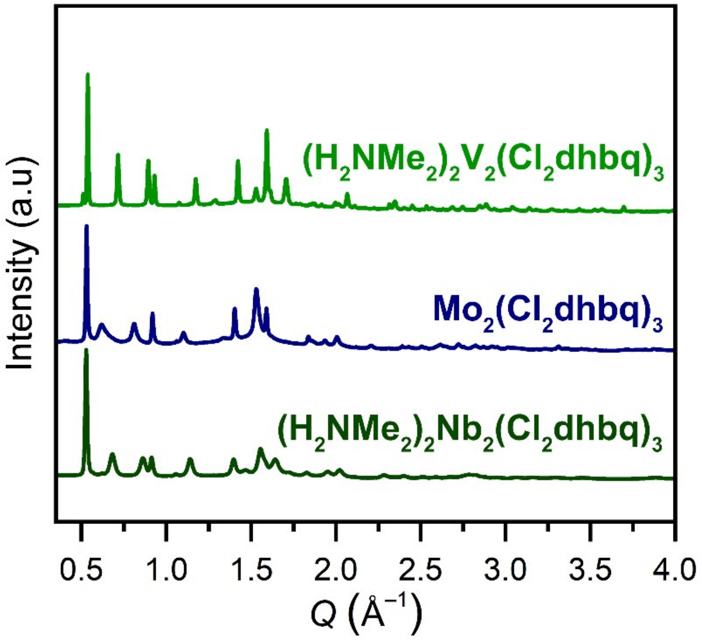

Figure S1. Comparison of powder X-ray diffraction of **1-Nb** (dark green), **2-Mo** (dark blue), and the previously reported vanadium phase $(H_2NMe_2)_2V_2(Cl_2dhbq)_3$.

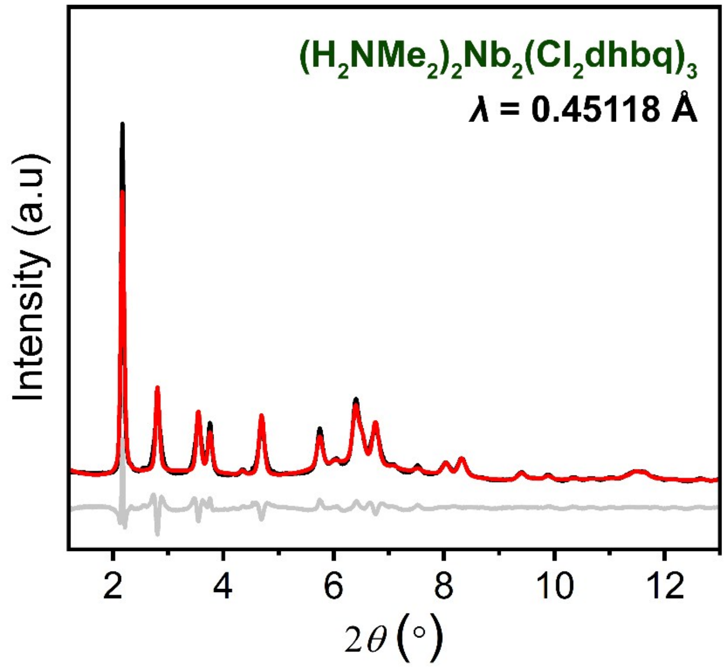

Figure S2. Le Bail refinement of powder X-ray diffraction data for **1-Nb**. Large differences in peak widths for *ab* reflections and *c* reflections limited the ability to accurately model peak profiles, which resulted in a relatively high R_{wp} (5.906%) and GoF (3.200) parameters. However, unit cell parameters obtained from peak indexing are in good agreement with the refined unit cell parameters, and only a small impurity peak is observed near $2\theta = 2.57°$, suggesting no lowering of symmetry. The refined unit cell parameters for the space group $P{-}31m$ are a = 13.743(6) Å and c = 9.204(8) Å. We note that $P{-}31m$ is not a unique space group for powder X-ray diffraction data, but this space group is assigned based on structurally analogous frameworks. We note that inclusion of the observed impurity peak in unit cell indexing did not generate a realistic alternative for the assigned space group.

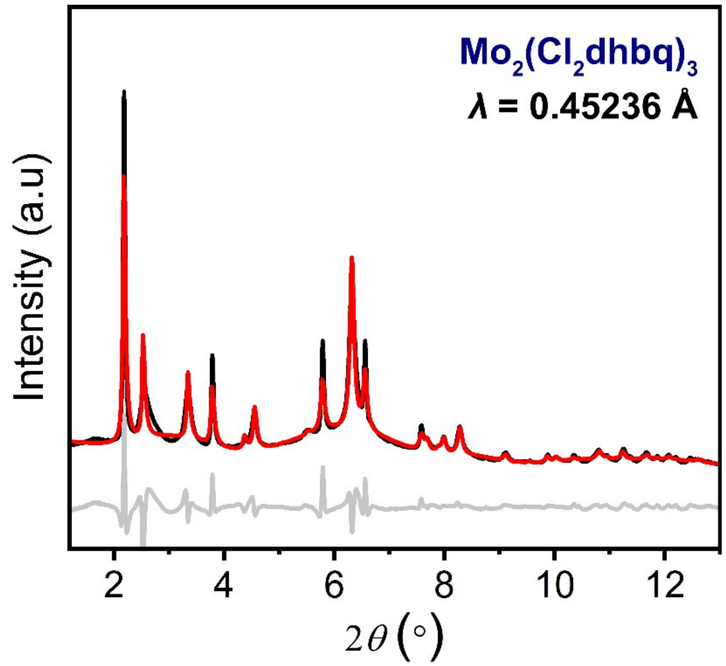

Figure S3. Le Bail refinement of powder X-ray diffraction data for **2-Mo**. Large differences in peak widths for *ab* reflections and *c* reflections (and a possible distribution of *c*-axis lattice spacings) limited the ability to accurately model peak profiles, which resulted in a relatively high R_{wp} (6.561%) and GoF (6.433) parameters. However, unit cell parameters obtained from peak indexing are in reasonable agreement with the refined unit cell parameters, and no peaks indicating a lowering of symmetry are observed. The refined unit cell parameters for the space group *P*−31*m* are *a* = 13.89(2) Å and *c* = 10.38(4) Å. We note that *P*−31*m* is not a unique space group for powder X-ray diffraction data, but this space group is assigned based on structurally analogous frameworks.

Spectroscopic Characterization

Discussion of XAS Oxidation State Assignments. In general, the effects of covalency on the *K*-edge energies are much more prominent for second row transition metals, which generally prevents absolute assignment of oxidation state in the absence of a well-defined model complex with similar coordination sphere.[5] To the best of our knowledge, there are no homoleptic niobium tris(dioxolene) complexes. While there are structurally analogous molybdenum tris(dioxolene) complexes, the Mo oxidation state in these complexes is not well-established. Spectra are reported for molybdenum tris(dithiolene) complexes, but the much more electron-donating nature of these ligands renders direct comparisons inappropriate. We note that in tris(dithiolene) complexes, oxidation state is largely an electron counting formalism, such that rising edge energies may not align well with formal oxidation states.[6]

K-edge spectra of metal oxides are frequently used for oxidation state comparison. However, changes in covalency, electron itinerancy, and connectivity, particularly for second- and third-row transition metal oxides, can strongly affect their rising edge energies. For example, the rising edge energy of **2-Mo** is substantially higher energy than that of MoO_2 (20015.3 eV versus 20010.9 eV, respectively), which could potentially indicate an oxidation state higher than Mo^{IV} in **2-Mo**. However, the metallic nature of MoO_2 may result in a reduced *K*-edge energy (lower effective oxidation state) due to strong metal–ligand covalency and metal–metal bonding. Semiconducting NbO_2 likely possesses reduced covalency and lacks metal–metal bonding, which may explain its higher rising edge energy relative to **1-Nb**. While the rising edge energy of **2-Mo** is far closer to that of MoO_3,[7] the rising edge energy for high-valent molybdenum oxides is heavily affected by the presence of a short Mo=O bond. Oxo complexes often show substantial shifts in their rising edge to lower energy relative to other complexes with similar oxidation state. Consequently, comparison to molybdenum oxides may not be appropriate for oxidations state assignment in **2-Mo**.

The difference in the rising edge energies of $NbCl_4$(THF) and **1-Nb** is 4.1 eV, while the difference in rising edge energies of $MoCl_4(CH_3CN)_2$ and **2-Mo** is 3.1 eV. The similar relative shifts for Nb and Mo likely suggest similar oxidation assignments for the two metals, though changes in the ligand redox states could complicate this assignment. As discussed in the text, given that the rising edge energy for **1-Nb** is between the rising edge energies of $NbCl_4$ and NbO_2, an assignment of Nb^{IV} seems most probable, and this seems consistent with other data discussed within the text. An assignment of Mo^{IV} for **2-Mo** is also consistent with other data. Thus, while the XAS data alone presents some ambiguity, the combined spectroscopic and magnetic data presented here support the redox-state assignments described in the text.

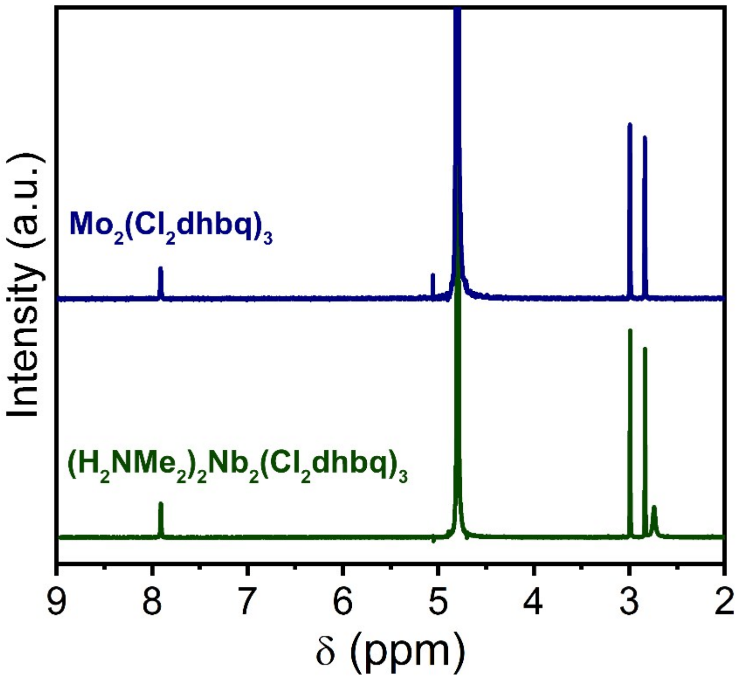

Figure S4. ^{1}H NMR spectra of **1-Nb** and **2-Mo** digested in D_2O. Both frameworks contain DMF. The peak at δ = 2.74 ppm in the **1-Nb** spectrum corresponds to dimethylammonium cations.

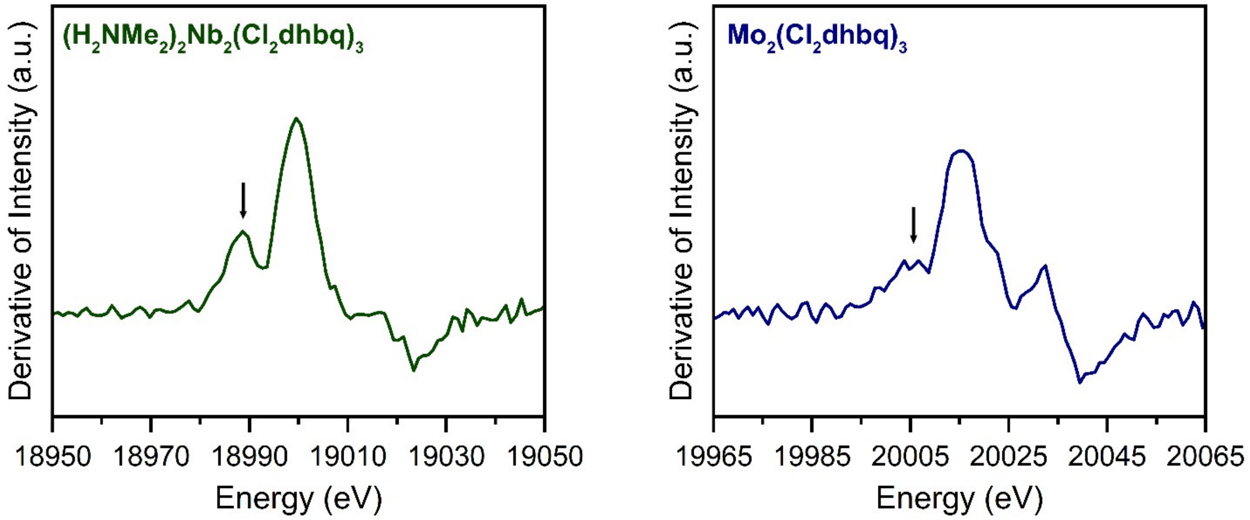

Figure S5. First derivatives of metal *K*-edge X-ray absorption spectra for **1-Nb** (left) and **2-Mo** (right). Pre-edge features indicative of a trigonal prismatic coordination geometry are marked with an arrow.

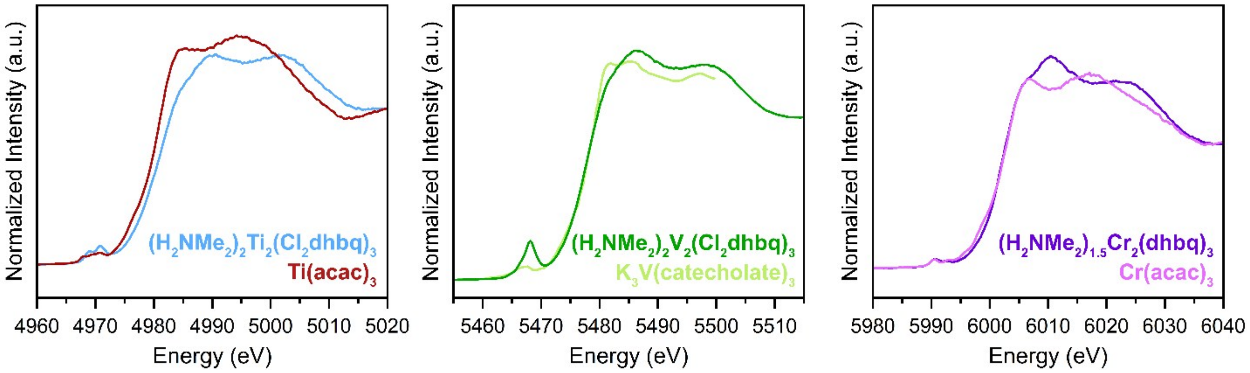

Figure S6. Metal *K*-edge X-ray absorption spectra for the titanium (left), vanadium (center), and chromium (right) congeners[8] of **1-Nb** and **2-Mo**. Only the vanadium congener displays a pre-edge feature consistent with a trigonal prismatic coordination geometry. Data for $K_3V(cathecholate)_3$ was taken from ref. 9.

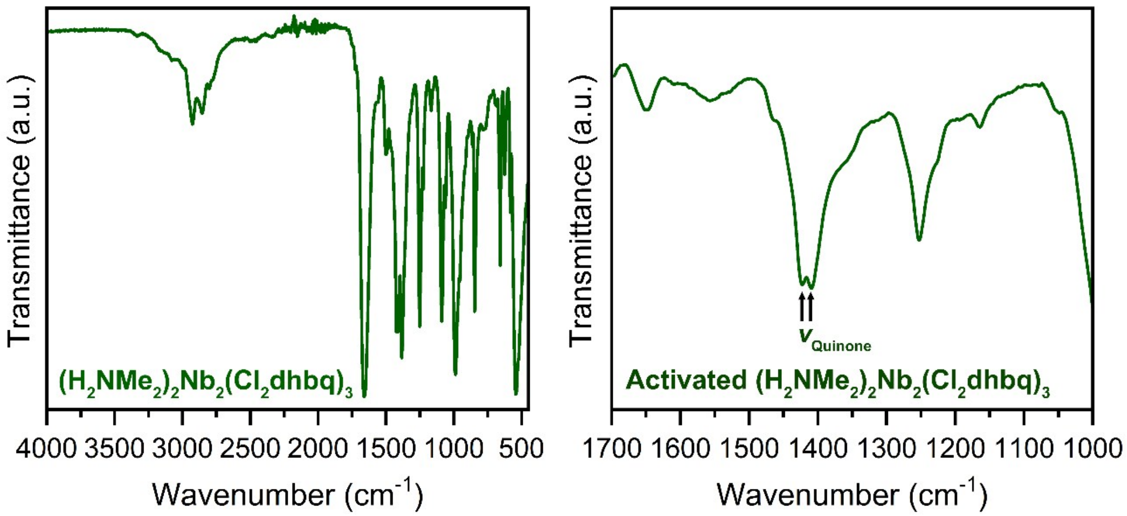

Figure S7. Full infrared spectrum of **1-Nb** (left) and magnified infrared spectrum of activated **1-Nb** (right). The presence of two quinone stretching frequencies in the activated framework confirms both features are intrinsic to the framework.

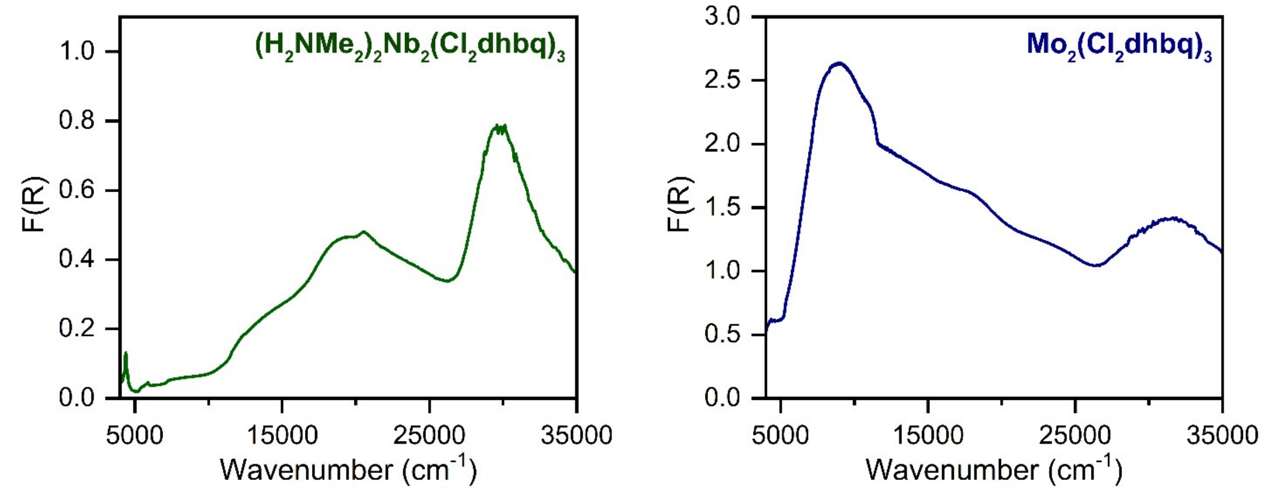

Figure S8. Diffuse reflectance UV-vis-NIR spectra of **1-Nb** (left) and **2-Mo** (right) diluted in $BaSO_4$. F(R) is the Kubelka-Munk transformation of the raw diffuse reflectance spectrum.

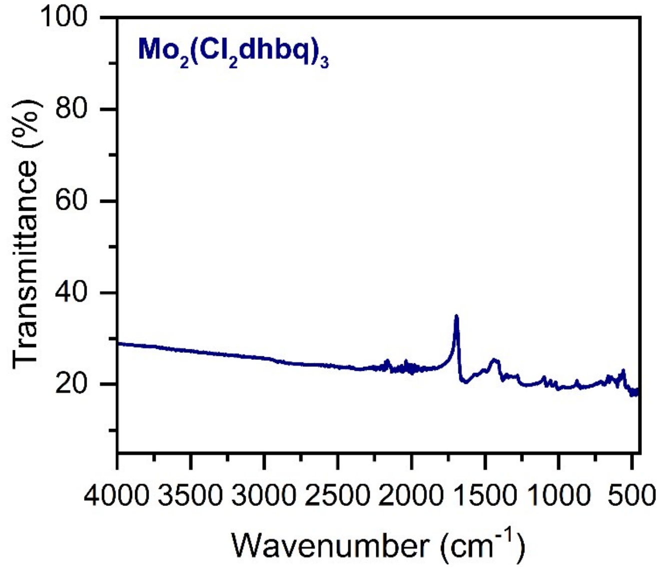

Figure S9. Full mid-infrared spectrum of **2-Mo**. A broad absorption masks the presence of any vibrational modes, which is typically indicative of free carriers.

Magnetic Characterization of 1-Nb and 2-Mo

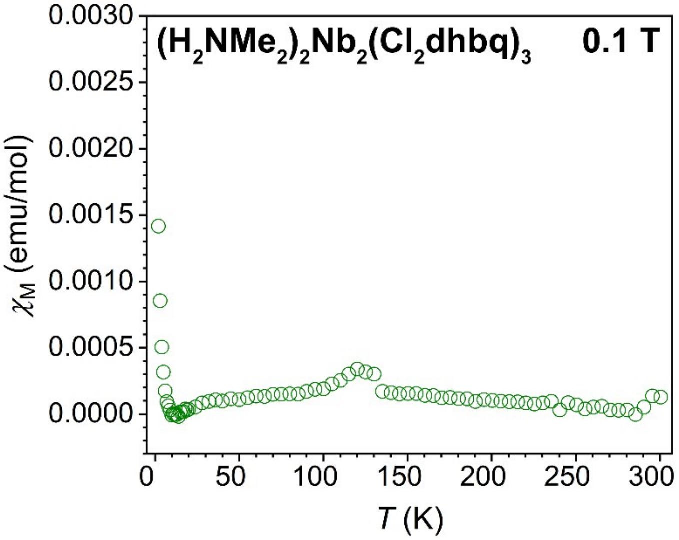

Figure S10. Zero field cooled dc magnetic susceptibility versus temperature for **1-Nb** at an applied field of 0.1 T. This measurement indicates the sample is effectively diamagnetic besides a small Curie tail below 5 K. Note that noise in the data and the anomaly near 130 K are due to an imperfect background subtraction and challenges associated with measuring low moment samples. Repeat measurements on this sample and on an independently prepared sample consistently showed small anomalies at different temperatures.

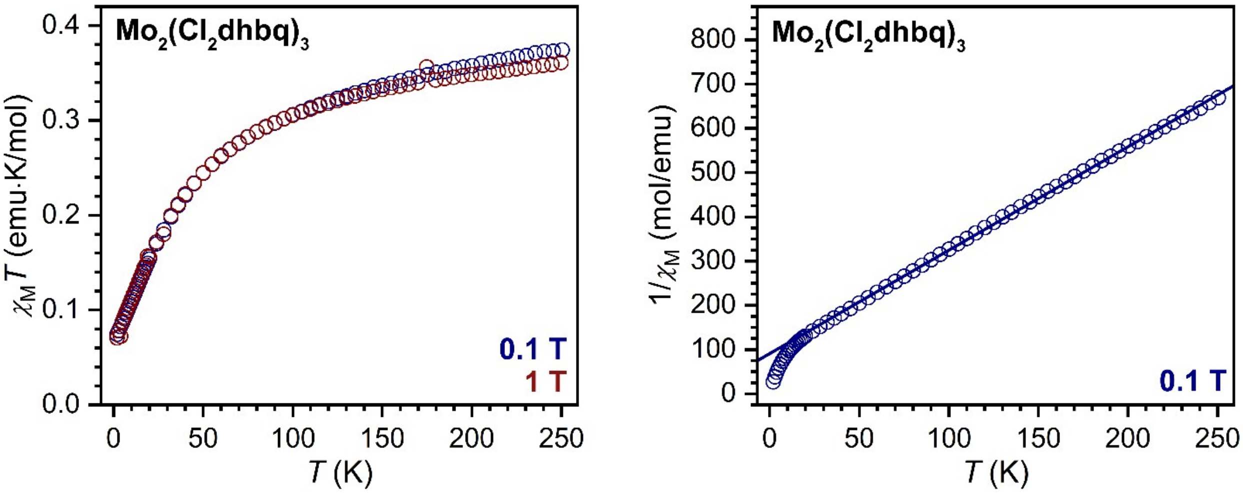

Figure S11. Left: Field cooled dc magnetic susceptibility times temperature versus temperature for **2-Mo** at applied fields of 0.1 and 1 T. No divergence was observed between zero-field-cooled and field-cooled data. Right: Curie-Weiss plot of inverse magnetic susceptibility versus temperature. A linear fit to the high temperature data yields Curie-Weiss parameters of $C = 0.43$ emu/mol·K and $\theta = -39$ K.

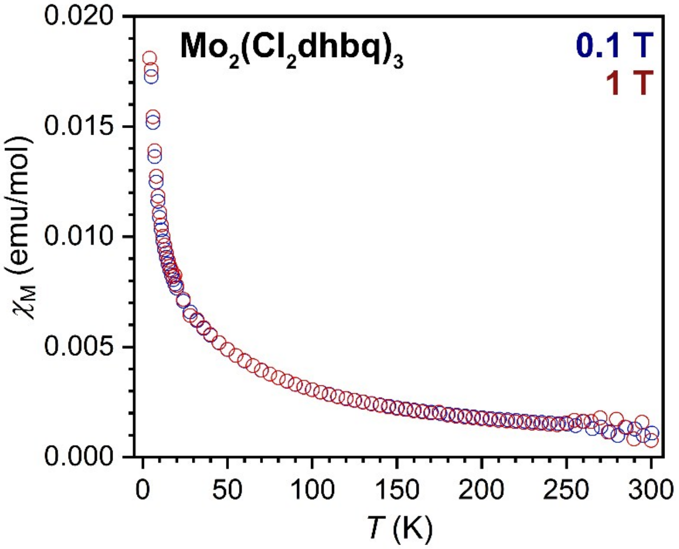

Figure S12. Field cooled dc magnetic susceptibility versus temperature for **2-Mo** at applied fields of 0.1 and 1 T over the temperature range 2–300 K. The comparable magnitude of the paramagnetic and diamagnetic susceptibilities of the sample above 250 K lead to increased measurement error and noisy data in this temperature range. However, no clear magnetic transition occurs in this temperature range, suggesting that the magnetic behavior below 250 K is representative of the data up to room temperature.

Electronic Conductivity

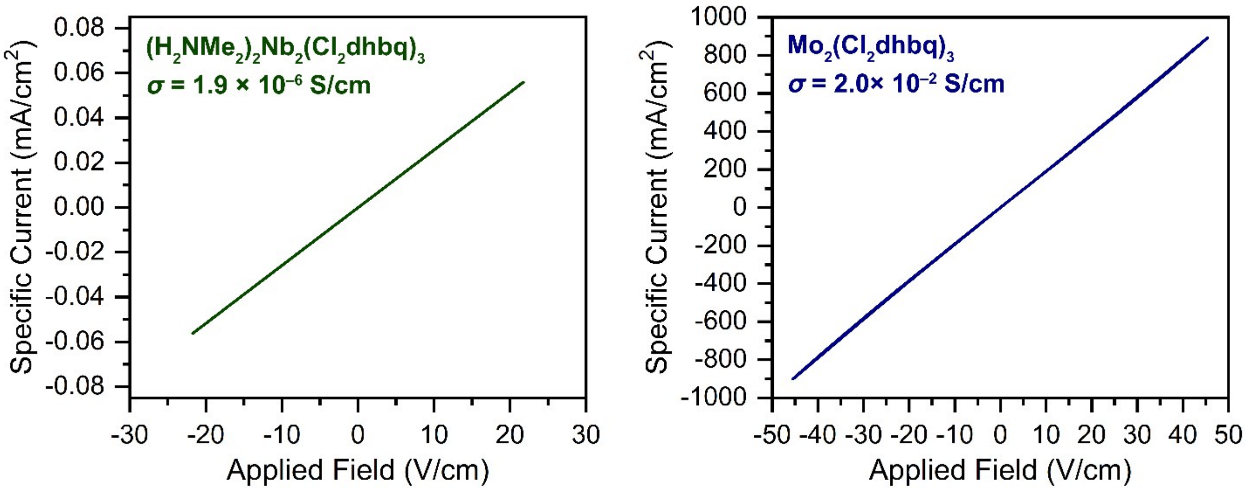

Figure S13. Linear current-voltage profiles for representative pressed-pellet conductivity measurements of **1-Nb** and **2-Mo**. Conductivity values reported in the main manuscript represent the average of three independent measurements.

Redox Activity of 1-Nb and 2-Mo

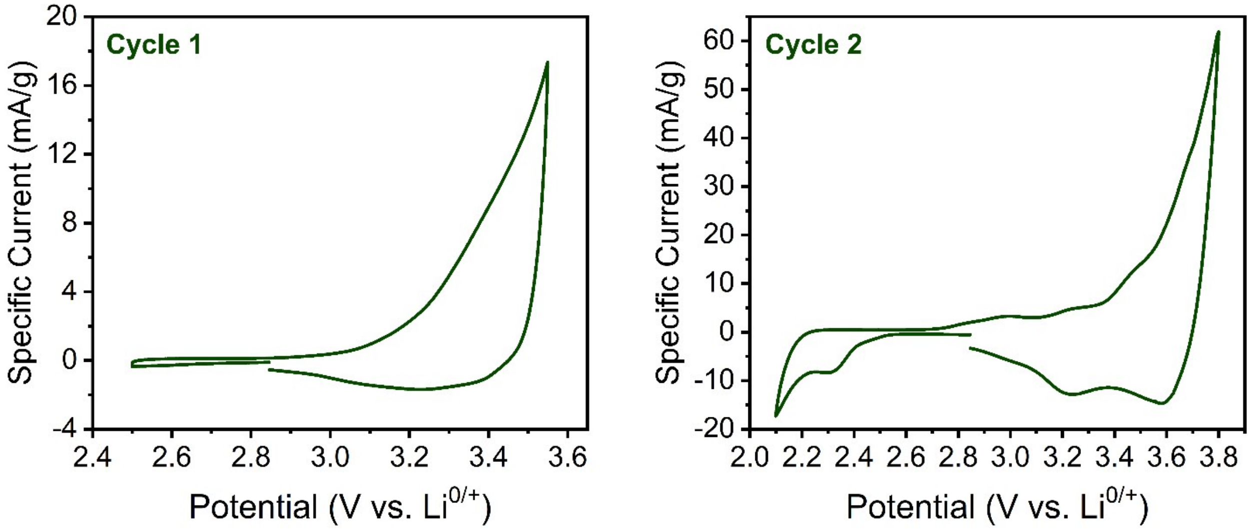

Figure S14. First two cycles of a solid-state cyclic voltammogram of **1-Nb**. Scans were collected at 50 μV/s with 0.1 M $LiBF_4$/propylene carbonate as the electrolyte. Data were collected starting with reduction.

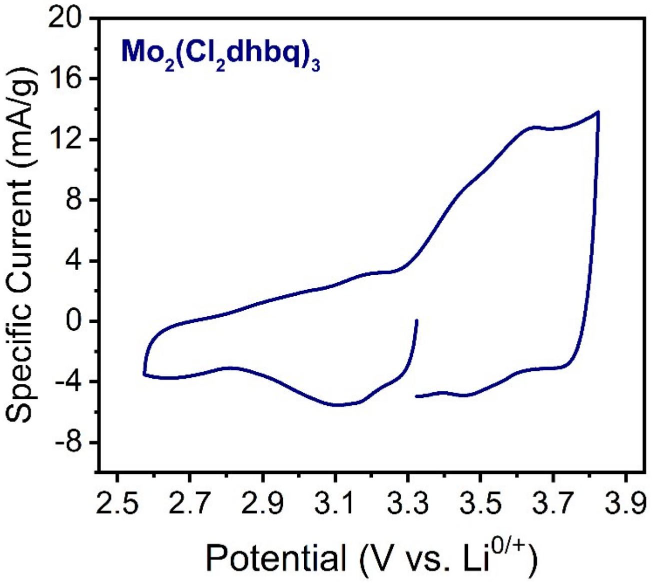

Figure S15. Solid-state cyclic voltammogram of **2-Mo** collected at 50 μV/s with 0.1 M $LiBF_4$/propylene carbonate as the electrolyte. Data were collected starting with reduction.

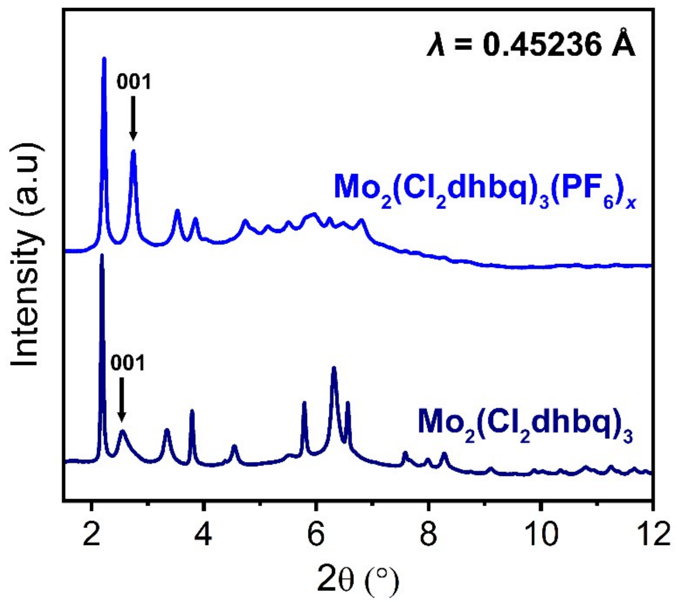

Figure S16. Powder X-ray diffraction data of **2-Mo** (bottom, dark blue) and **2-Mo** oxidized with ferrocenium hexafluorophosphate (top, light blue) collected at $\lambda = 0.45236$ Å. The narrower peak width for the 001 reflection compared to as-synthesized **2-Mo** likely suggests improved stacking order or longer particle dimensions in the *c* direction, both of which could be caused by oxidative insertion of hexafluorophosphate.

Exfoliation of 2-Mo

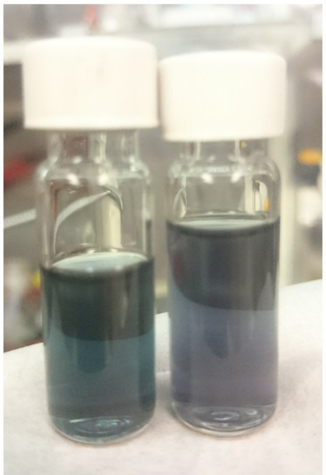

Figure S17. Photograph depicting colloidal suspensions of **2-Mo** in DMF (left) and CH_3CN (right).

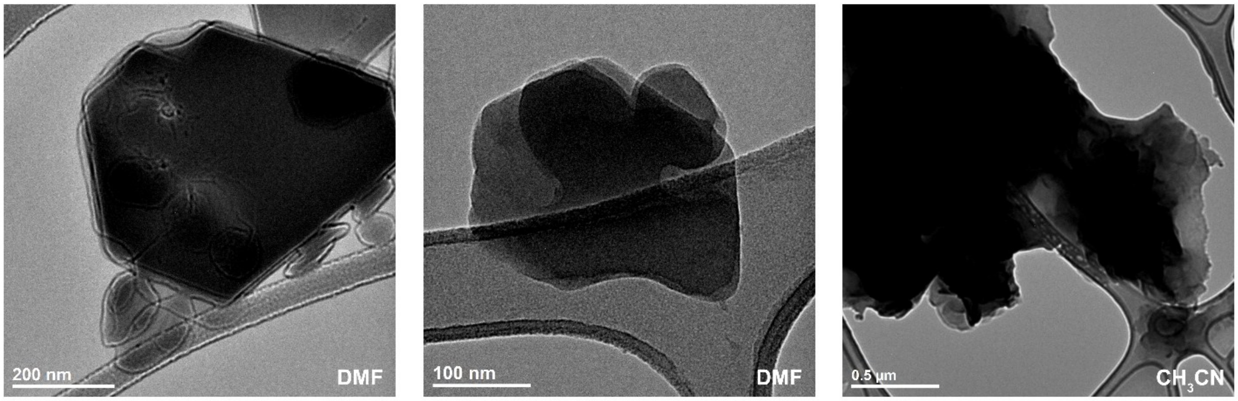

Figure S18. Representative TEM images of nanosheets of **2-Mo** in DMF(left, center) and CH_3CN (right). Many nanosheets exfoliated in DMF displayed 100–250 nm edges and hexagonal angles, in addition to less well-defined particle shapes. Nanosheets exfoliated in acetonitrile aggregated far more than the DMF-exfoliated nanosheets, but thin layers could be observed near the edges of some aggregates.

References

(1) A.A. Coelho, *J. Appl. Cryst*., 2003, **36**, 86.

(2) A.A. Coelho, TOPAS-Academic, version 4.1. 2007.

(3) V. Favre-Nicolin, R. Cerny, *J. Appl. Cryst.,* 2002, **35**, 734-743.

(4) V. Favre-Nicolin, R. Cerny, FOX, Free Objects for Crystallography. http://objcryst.sourceforge.net.

(5) F. A. Lima, R. Bjornsson, T. Weyhermüller, P. Chandrasekaran, P. Glatzel, F. Neese and S. DeBeer, *Phys. Chem. Chem. Phys.*, 2013, **15**, 20911–20920.

(6) R. R. Kapre, E. Bothe, T. Weyhermüller, S. D. B. George and K. Wieghardt, *Inorg. Chem.*, 2007, **46**, 5642–5650.

(7) M. Lv, W. Xie, S. Sun, G. Wu, L. Zheng, S. Chu, C. Gao and J. Bao, *Catal. Sci. Technol.*, 2015, **5**, 2925–2934.

(8) M. E. Ziebel, L. E. Darago, J. R. Long. *J. Am. Chem. Soc*. 2018, **140**, 3040-3051.

(9) P. Franl, K. O. Hodgson. *Inorg. Chem*. 2000, **39**, 6018-6027.
